# Supplementary material for: The moderating role of extraversion in the relationship between trait mindfulness and pain adaptation
Source: Front Pain Res (Lausanne). 2025 Apr 1;6:1534339. doi: 10.3389/fpain.2025.1534339 (PMC11996774; doi:10.3389/fpain.2025.1534339)
Supplement: Supplementary file 1 [file Table1.docx]

**Supplementary Material**

Chen Lu, Nele Berner, Lena Hagel, Nils Jannik Heukamp, Vera Moliadze*, Frauke Nees*

Institute of Medical Psychology and Medical Sociology, University Medical Center Schleswig-Holstein, Kiel University, Kiel, Germany

*Shared last authorship

**Correspondence should be addressed to**: Frauke Nees, Institute of Medical Psychology and Medical Sociology, University Medical Center Schleswig-Holstein, Kiel University, Preußerstraße 1-9 24105 Kiel, Germany.

E-mail: nees@med-psych.uni-kiel.de

Tel: +49 431 / 500-30800

Fax: +49 431 / 500-30804

**1. The Results of Analysis** **when Using the Difference between the First and Tenth Trials as the Indicator of Pain Adaptation.**

***1.1 Correlations between Variables.***

As shown in Table S1 and Table S2, when using the difference between the first and tenth trials as the indicator of pain adaptation, the correlations between variables were consistent with when using the first and fifteenth trials as the indicator, no matter whether controlling for sex or not.

There is a significant positive correlation (r = 0.783, p < 0.001) between the two assessments of pain adaptation (the difference between the first and fifteenth trials VS. the difference between the first and tenth trials of pain intensity ratings).

***1.2 The Predictive Value of Trait Mindfulness and Extraversion for Pain Adaptation.***

As shown in Table S3, when using the difference between the first and tenth trials as the indicator of pain adaptation, the non-significant prediction of trait mindfulness and extraversion on pain adaptation were consistent with when using the first and fifteenth trials as the indicator, no matter whether controlling for sex or not.

***1.3 The Moderation of the Relationship between Trait Mindfulness and Pain Adaptation by Extraversion.***

As shown in Model 3 (see Table S3), there was a significant interaction between trait mindfulness and extraversion (β = -0.314, p = 0.026) which was consistent with the findings when the first and fifteenth trials as the indicator of pain adaptation.

Applying the simple slope analysis (see Figure S1), we see that at a lower level of extraversion, a higher level of trait mindfulness didn’t significantly predict a larger degree of pain adaptation (b = 3.543, p = 0.392), which was found to be significant when the first and fifteenth trials as the indicator of pain adaptation. However, what was consistent with the findings when using the first and fifteenth trials as the indicator of pain adaptation was, at a higher level of extraversion, a higher level of trait mindfulness significantly predicted a lesser degree of pain adaptation (b = -8.160, p = 0.043), and at a medium level of extraversion, a higher level of trait mindfulness did not significantly predict a lesser degree of pain adaptation (b = -2.309, p = 0.461).

As shown in Figure S2, the effect of trait mindfulness was significant at values of extraversion above 0.400. Outside of this range, the effect was not significant.

| **Table S1. Correlations of Variables.** | | | | | | | |
| --- | --- | --- | --- | --- | --- | --- | --- |
| **Variables** | **M ± SD** | **Observed Range** | **Trait Mindfulness (MAAS)** | **Extraversion (NEO-FFI)** | **Pain Adaptation** | **Pain Stimulus Temperature** | **Sex** |
| **Trait Mindfulness (MAAS)** | **3.76 ± 0.739** | **2.20 ~ 4.87** | **1** |  |  |  |  |
| **Extraversion (NEO-FFI)** | **2.43 ± 0.443** | **1.50 ~ 3.33** | **0.347^*^** | **1** |  |  |  |
| **Pain Adaptation** | **3.64 ± 14.903** | **-29.81 ~ 46.30** | **-0.035** | **0.056** | **1** |  |  |
| **Pain Stimulus Temperature** | **46.64 ± 1.921** | **38.80 ~ 49.00** | **0.103** | **0.072** | **0.022** | **1** |  |
| **Sex** | **female** = **0 male** = **1** |  | **0.356^**^** | **0.114** | **0.155** | **0.277^*^** | **1** |
| **Age** | **23.29 ± 2.052** | **20 ~ 30** | **-0.163** | **-0.002** | **-0.070** | **-0.164** | **0.148** |

***** p < 0.05, ****** p < 0.01

Abbreviations: M, Mean; MAAS, Mindful Attention and Awareness Scale; NEO-FFI, NEO Five Factors Inventory; SD, Standard Deviation.

| **Table S2. Correlations of Variables When Controlling for Sex.** | | | | | |
| --- | --- | --- | --- | --- | --- |
| **Variables** | | **Trait Mindfulness (MAAS)** | **Extraversion (NEO-FFI)** | **Pain Adaptation** | **Pain Stimulus Temperature** |
| **Sex (Control Variable)** | **Trait Mindfulness (MAAS)** | **1** |  |  |  |
|  | **Extraversion (NEO-FFI)** | **0.330*** | **1** |  |  |
|  | **Pain Adaptation** | **-0.097** | **0.039** | **1** |  |
|  | **Pain Stimulus Temperature** | **0.005** | **0.042** | **-0.022** | **1** |
|  | **Age** | **-0.233** | **-0.020** | **-0.095** | **-0.216** |

***** p < 0.05

Abbreviations: M, Mean; MAAS, Mindful Attention and Awareness Scale; NEO-FFI, NEO Five Factors Inventory; SD, Standard Deviation.

**Table S3. Hierarchical Regression Analysis Predicting Pain Adaptation from Trait Mindfulness, Extraversion, and the Mindfulness × Extraversion Interaction.**

| **Variable** | **Model 1 (Predictors)** | **Model 2 (Predictors and Control Variable)** | **Model 3 (Moderation Model)** |
| --- | --- | --- | --- |
| **Sex (female = 0, male = 1)** |  | **5.893 (0.192)** | **6.854 (0.224)** |
| **Trait Mindfulness** | **-1. 241 (-0.062)** | **-2.635 (-0.131)** | **-2.315 (-0.115)** |
| **Extraversion** | **2.597 (0.077)** | **2.663 (0.079)** | **2.408 (0.072)** |
| **Trait Mindfulness × Extraversion** |  |  | **-13.212 (-0.314) *** |
| **R²** | **0.006** | **0.039** | **0.136** |
| **ΔR²** | **0.006** | **0.032** | **0.097** |
| **F** | **0.159** | **0.645** | **1. 848** |
| **df** | **2, 49** | **3, 48** | **4, 47** |

***** p **< 0.05**

All continuous independent variables were centered (original values minus mean) before being entered into the models. Outside the brackets are the B values (unstandardized coefficient) and inside the brackets are the β values (standardized coefficient).

**Figure S1.**

**The Relationship between Trait Mindfulness and Pain Adaptation at Three Levels of Extraversion.**

All continuous independent variables were centered (original values minus mean) before being entered into the models and the units for each variable were one point. The pain adaptation assessed by the difference between the first trials and the tenth trials of ratings. The relationship between trait mindfulness and pain adaptation was examined on three levels of extraversion and levels of extraversion were defined by one standard deviation below the mean, mean, and one standard deviation above the mean.

**Figure S2.**

**
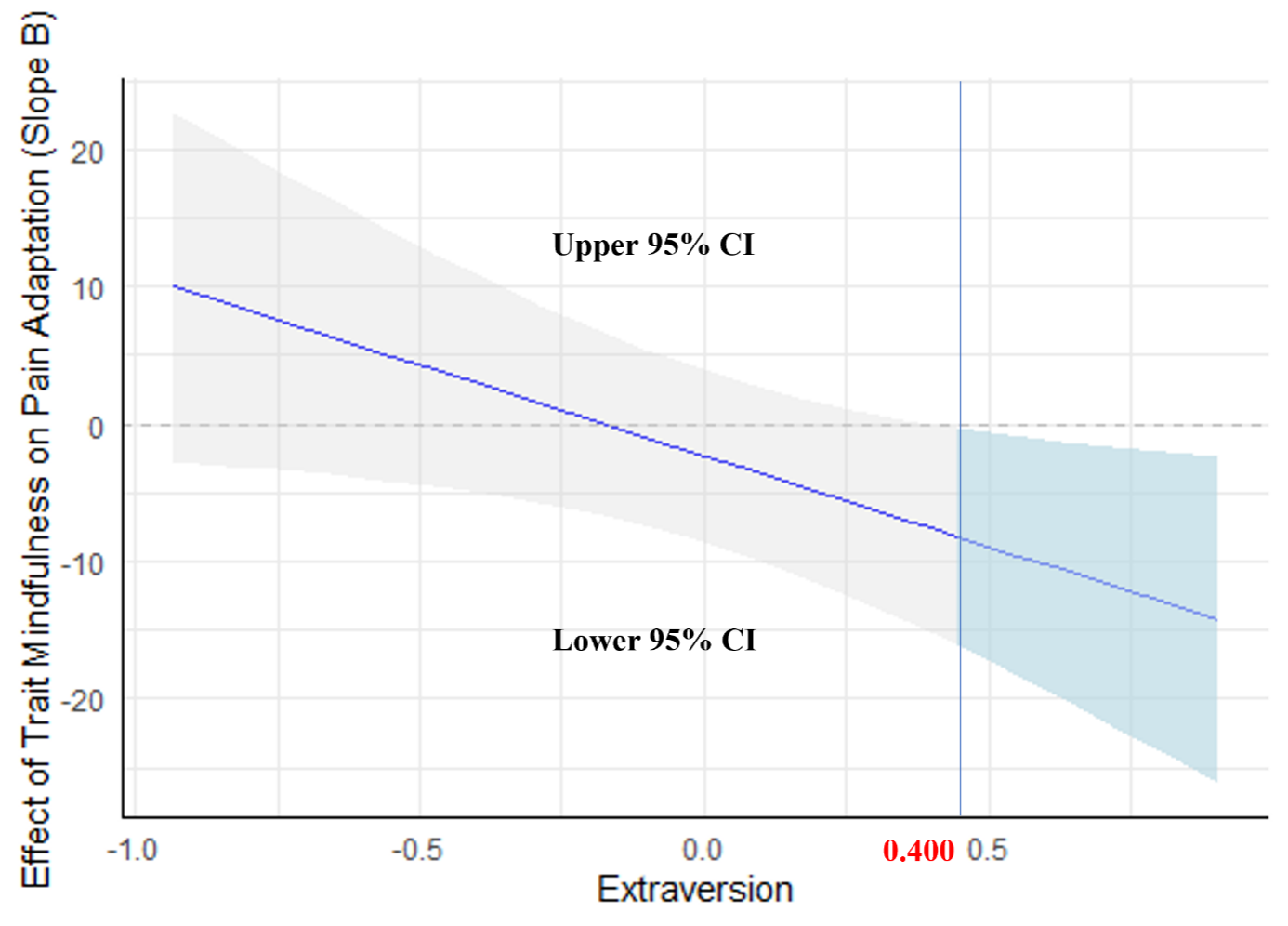
**

**Johnson-Neyman plot of the conditional effect of Trait mindfulness on Pain Adaptation across Extraversion.**

All continuous independent variables were centered (original values minus mean) before being entered into the models and the units for each variable was one point. The pain adaptation was assessed by the difference between the first trials and the tenth trials of ratings. The grey shaded area indicates the confidence interval (CI), and the light blue shaded area indicates the significance area.

**2. Results of the Tests for the Regression Model's Assumptions.**

*2.1 Using the Difference between the First and Fifteenth Trials as the Indicator of Pain* *Adaptation.*

As shown in Figure S3 and Figure S4, the normality of residuals was evaluated visually using a histogram of standardized residuals and a probability-probability plot (P-P plot). Both plots indicated that the residuals approximately followed a normal distribution.

As shown in Figure S5, variance homogeneity was examined through a scatterplot of standardized residuals against predicted values. The residual plot showed no discernible pattern, suggesting constant variance across levels of the predictor variables.

Autocorrelation was assessed using the Durbin-Watson statistic, with values = 2.050, near 2, indicating no significant first-order autocorrelation.

As shown in Table S5, multicollinearity was evaluated by calculating both the tolerance and the variance inflation factor (VIF) for each predictor variable. All tolerance values were above 0.1, and all VIF values were well below the threshold of 10, indicating a low risk of multicollinearity.

A casewise diagnostic was also performed, identifying observations with standardized residuals exceeding three standard deviations to detect influential outliers; no such cases were found, suggesting the model is robust and not unduly affected by extreme values. Together, these diagnostics confirm that the model satisfies the fundamental assumptions, supporting the reliability of the regression estimates.

**Figure S3.**


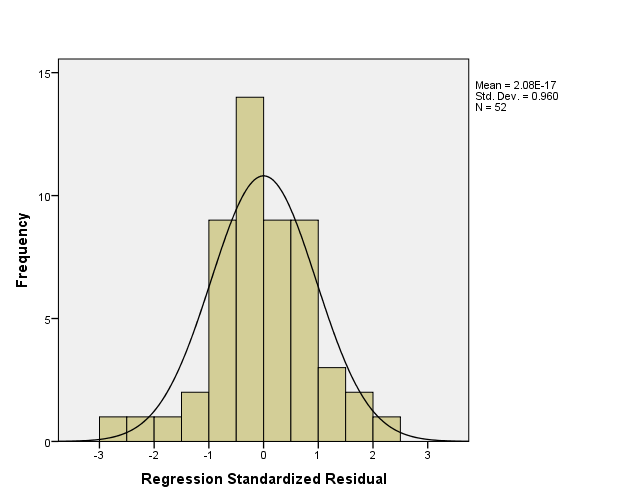


**Histogram of Standardized Residuals**

**The dependent variable is the pain adaptation which is assessed from the difference between the first trial and the fifteenth trial of the pain intensity ratings.**

**Figure S4**

**
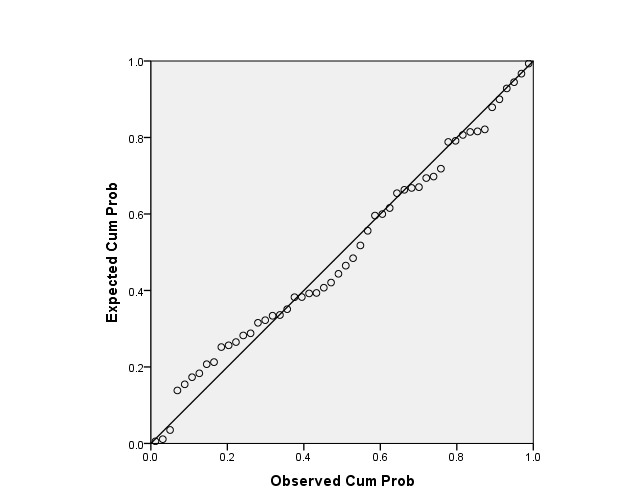
**

**Normal P-P Plot of Regression Standardized Residual.**

The dependent variable is the pain adaptation which is assessed from the difference between the first trial and the fifteenth trial of the pain intensity ratings.

**Figure S5**


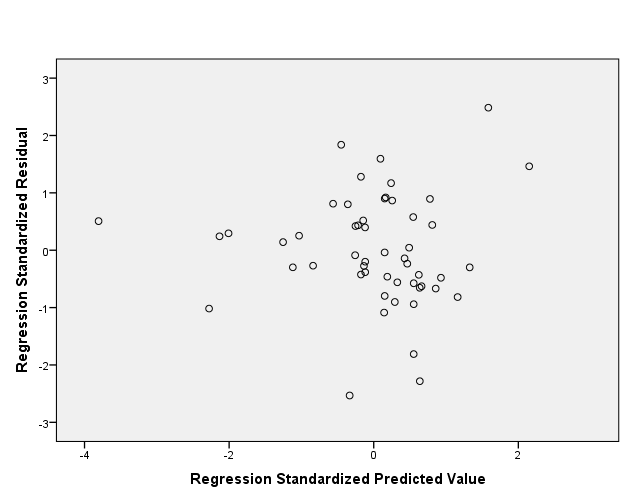


**Scatterplot.**

The dependent variable is the pain adaptation assessed from the difference between the first trial and the fifteenth trial of the pain intensity ratings.

| **Table S5. The Tolerance and the Variance Inflation Factor for Each Predictor Variable.** | | | |
| --- | --- | --- | --- |
| **Model** | | **Collinearity Statistics** | |
|  |  | **Tolerance** | **VIF** |
| **1** | **Trait Mindfulness** | **0.880** | **1.137** |
|  | **Extraversion** | **0.880** | **1.137** |
| **2** | **Trait Mindfulness** | **0.779** | **1.284** |
|  | **Extraversion** | **0.880** | **1.137** |
|  | **Sex** | **0.873** | **1.145** |
| **3** | **Trait Mindfulness** | **0.777** | **1.287** |
|  | **Extraversion** | **0.879** | **1.137** |
|  | **Sex** | **0.866** | **1.155** |
|  | **Trait Mindfulness × Extraversion** | **0.985** | **1.016** |

The dependent variable is the pain adaptation assessed from the difference between the first trial and the fifteenth trial of the pain intensity ratings. All continuous independent variables were centered (original values minus mean) before being entered into the models.

*2.2 Using the Difference between the First and Tenth Trials as the Indicator of Pain* *Adaptation.*

As shown in Figure S6 and Figure S7, the normality of residuals was evaluated visually using a histogram of standardized residuals and a probability-probability plot (P-P plot). Both plots indicated that the residuals approximately followed a normal distribution.

As shown in Figure S8, variance homogeneity was examined through a scatterplot of standardized residuals against predicted values. The residual plot showed no discernible pattern, suggesting constant variance across levels of the predictor variables.

Autocorrelation was assessed using the Durbin-Watson statistic, with values = 1.745, near 2, indicating no significant first-order autocorrelation.

As shown in Table S6, multicollinearity was evaluated by calculating both the tolerance and the variance inflation factor (VIF) for each predictor variable. All tolerance values were above 0.1, and all VIF values were well below the threshold of 10, indicating a low risk of multicollinearity.

A casewise diagnostic was also performed, identifying observations with standardized residuals exceeding three standard deviations to detect influential outliers; no such cases were found, suggesting the model is robust and not unduly affected by extreme values. Together, these diagnostics confirm that the model satisfies the fundamental assumptions, supporting the reliability of the regression estimates.

**Figure S6**


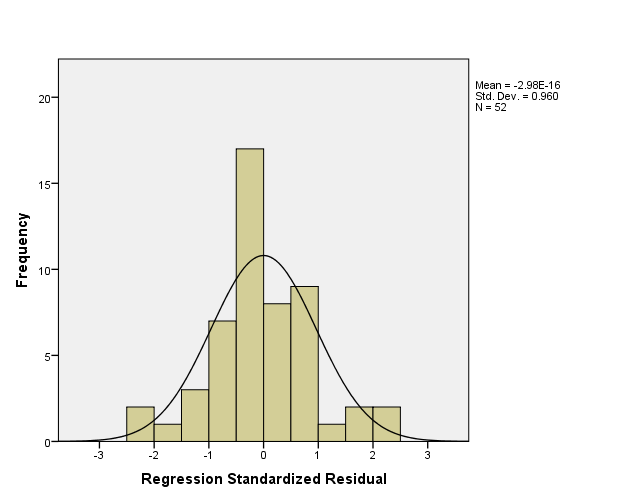


**Histogram of Standardized Residuals**

The dependent variable is the pain adaptation assessed from the difference between the first trial and the tenth trial of the pain intensity ratings.

**Figure S7**


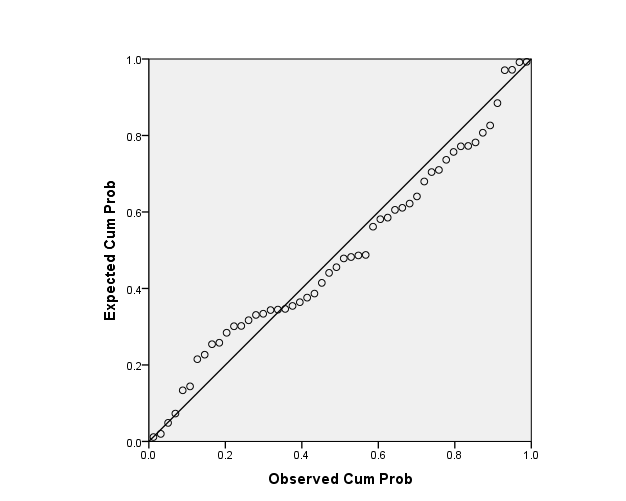


**Normal P-P Plot of Regression Standardized Residual.**

The dependent variable is the pain adaptation assessed from the difference between the first trial and the tenth trial of the pain intensity ratings.

**Figure S8**


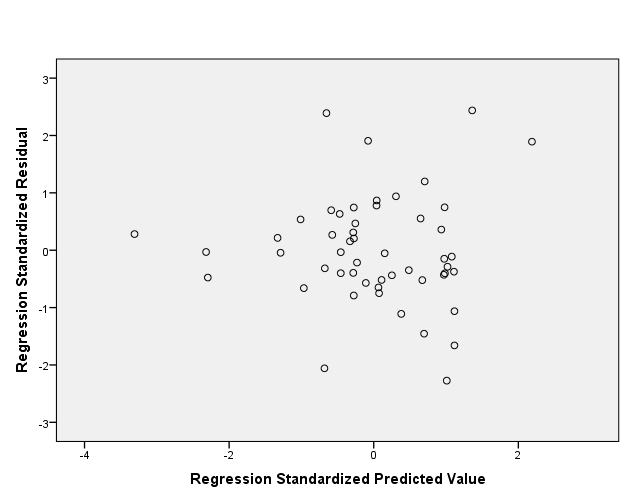


**Scatterplot.**

The dependent variable is the pain adaptation which is assessed from the difference between the first trial and the tenth trial of the pain intensity ratings.

| **Table S6. The Tolerance and the Variance Inflation Factor for Each Predictor Variable.** | | | |
| --- | --- | --- | --- |
| **Model** | | **Collinearity Statistics** | |
|  |  | **Tolerance** | **VIF** |
| **1** | **Trait Mindfulness** | **0.880** | **1.137** |
|  | **Extraversion** | **0.880** | **1.137** |
| **2** | **Trait Mindfulness** | **0.779** | **1.284** |
|  | **Extraversion** | **0.880** | **1.137** |
|  | **Sex** | **0.873** | **1.145** |
| **3** | **Trait Mindfulness** | **0.777** | **1.287** |
|  | **Extraversion** | **0.879** | **1.137** |
|  | **Sex** | **0.866** | **1.155** |
|  | **Trait Mindfulness × Extraversion** | **0.985** | **1.016** |

The dependent variable is the pain adaptation which is assessed from the difference between the first trial and the tenth trial of the pain intensity ratings. All continuous independent variables were centered (original values minus mean) before being entered into the models.
