# Supplementary material for: The moderating role of extraversion in the relationship between trait mindfulness and pain adaptation
Source: Front Pain Res (Lausanne). 2025 Apr 1;6:1534339. doi: 10.3389/fpain.2025.1534339 (PMC11996774; doi:10.3389/fpain.2025.1534339)
Supplement: Supplementary file 3 [file Table3.docx]

**The Moderating Role of Extraversion in the Relationship between Trait Mindfulness and Pain Adaptation**

Chen Lu, Nele Berner, Lena Hagel, Nils Jannik Heukamp,

Vera Moliadze*, Frauke Nees*

Institute of Medical Psychology and Medical Sociology, University Medical Center Schleswig-Holstein, Kiel University, Kiel, Germany

*Shared last authorship

**Correspondence should be addressed to**: Frauke Nees, Institute of Medical Psychology and Medical Sociology, University Medical Center Schleswig-Holstein, Kiel University, Preußerstraße 1-9 24105 Kiel, Germany.

E-mail: nees@med-psych.uni-kiel.de

Tel: +49 431 / 500-30800

Fax: +49 431 / 500-30804

**Keywords:** extraversion, habituation, moderation model, pain adaptation, trait mindfulness.

DRKS-ID: DRKS00032271

**Abstract**

**Introduction:** Pain habituation, the reduction in response to repeated painful stimuli, is a positive adaptation process, while pain sensitization is linked to chronic pain. Traits like mindfulness and extraversion affect pain processing, but their influence on pain adaptation and potential interactions remains underexplored. This study aimed to examine the relationship between trait mindfulness, extraversion, and pain adaptation, assessing their predictive value and any interaction effects.

**Materials and Method:** Fifty-two healthy participants, mean age = 23.29 ± 2.052 years, completed questionnaires measuring trait mindfulness and extraversion, followed by an experimental pain stimulation to assess pain adaptation. Correlation analysis and hierarchical regression analysis were used to explore the relationships between traits and pain adaptation, and potential interaction effects.

**Result:** Trait mindfulness positively correlated with extraversion, but neither trait showed a significant correlation with pain adaptation. In addition, neither trait mindfulness nor extraversion significantly predicted pain adaptation. However, a significant interaction was found between the two traits, suggesting that extraversion moderates the relationship between trait mindfulness and pain adaptation.

**Conclusion:** Trait mindfulness and extraversion are interrelated traits whose interaction affects pain habituation and the extent to which individuals with higher levels of trait mindfulness exhibit greater pain adaptation appears to depend on their levels of extraversion. These findings suggest that trait mindfulness and extraversion may act as protective factors in chronic pain development. Mindfulness-based interventions may be particularly effective for individuals with specific personality traits. This can inform further research to explore these implications for pain management.

**1. Introduction**

Repeated painful stimulation usually causes a decreased neurophysiological and behavioral response (habituation), which is suggested to represent a positive adaptation process (Ginzburg et al., 2015), whilean increased response to pain (sensitization), is regarded as maladaptation in this adaptation process and is also discussed as a mechanism for pain chronicity (Bingel, Schoell, Herken, Büchel, & May, 2007; Defrin, Riabinin, Feingold, Schreiber, & Pick, 2015; van der Miesen, Vossen, & Joosten, 2023). This also relates to findings that the pain adaptation process is sensitive to various individual factors already in healthy individuals (Ginzburg et al., 2015; Harrison, Salomons, van Reekum, Adams, & Gandhi, 2023). Among these factors, trait-like individual tendencies might be particularly important, as they represent relatively stable characteristics that can serve as fundamental risk factors in predicting an individual's vulnerability to developing chronic pain. In this context, trait mindfulness has increasingly been discussed in recent years, also in the context of potential (mindfulness-based) interventions for chronic pain (Day, Jensen, Ehde, & Thorn, 2014; Day & Thorn, 2016; Kiken, Garland, Bluth, Palsson, & Gaylord, 2015; Shapiro, Brown, Thoresen, & Plante, 2011), and has been found to alter the experience of pain (Ciere et al., 2019; Kusko, 2023; Miniati et al., 2023). Trait mindfulness refers to an individual's characteristic tendency to maintain awareness of the present moment in a nonreactive and nonjudgmental manner (Carpenter, Conroy, Gomez, Curren, & Hofmann, 2019). Healthy individuals with higher compared to individuals with lower levels of trait mindfulness have been shown to report higher pain thresholds, less pain catastrophizing, and lower pain intensity and pain unpleasantness ratings when experiencing experimentally induced painful stimulation (Harrison, Zeidan, Kitsaras, Ozcelik, & Salomons, 2019; Zeidan et al., 2018).

Moreover, also personality traits such as extraversion have been shown to alter pain experience, e.g., higher levels of extraversion are linked to greater pain tolerance and higher pain thresholds while increased pain sensitivity (Ferracuti & De Carolis, 2005; Grouper, Eisenberg, & Pud, 2021; Phillips & Gatchel, 2000; Vassend, Røysamb, & Nielsen, 2013; Wade & Price, 2000). Extraversion refers to a personality dimension regarding individual differences in sociability, assertiveness, positive emotionality, approach tendencies, and status motivation (Lukaszewski, 2020).

Interestingly, trait mindfulness and extraversion are two related traits as found in previous research, however, some studies found a positive correlation between them, while others suggest their positive correlation was not significant or even negative (Banfi & Randall, 2022; Giluk, 2009; Hanley & Garland, 2017). Additionally, it remains unclear whether higher levels of trait mindfulness and extraversion facilitate the pain adaptation process in healthy individuals and how these are related in this respect. While lower levels of trait mindfulness were linked to pain sensitization (Harrison et al., 2023), suggesting a facilitating effect of trait mindfulness on the pain adaptation process, higher levels of trait mindfulness were found to be associated with incomplete habituation (refer to the pain rating higher than 0 at the end of the 60-second stimulus according to their experiment design) (Ginzburg et al., 2015), indicating a hindering effect of trait mindfulness on the pain adaptation process. These mixed findings suggest that experimental research is needed to determine whether trait mindfulness facilitates or impairs the pain adaptation process. In addition, experimental exploration of the relationship between extraversion and pain adaptation is also limited. Existing research indicates that individuals’ levels of extraversion affect their pain experience. For example, individuals with higher levels of extraversion have shown greater pain tolerance and higher pain thresholds (Ferracuti & De Carolis, 2005; Phillips & Gatchel, 2000). However, higher levels of extraversion have also been linked to increased pain sensitivity (Grouper et al., 2021). Moreover, it is also not clear whether extraversion is also a factor influencing pain adaptation, as well as, whether and how extraversion plays a significant role in the interaction of trait mindfulness and pain adaptation process. Although both extraversion (Diener, Suh, Lucas, & Smith, 1999) and trait mindfulness (Brown, Ryan, & Creswell, 2007) are associated with subjective well-being and positive emotions, extraversion is marked by a preference for activity, excitement, and positive stimuli (Costa & McCrae, 2008; HE, YUAN, WU, & LI, 2008), which may conflict with the practice of trait mindfulness. This conflict can make it difficult for extroverts to maintain mindfulness in the present moment, particularly during negative experiences, such as painful stimuli (Giluk, 2009; Grouper et al., 2021). Therefore, the proposed facilitative effect of higher levels of trait mindfulness on the pain adaptation process may depend on the level of extraversion.

Therefore, the present study aimed to examine the relationships between trait mindfulness, extraversion, and pain adaptation (measured through ratings of repeated painful stimulation). In addition, we examined the predictive value of trait mindfulness and extraversion for pain adaptation. Particularly, we were interested in whether extraversion represents a significant moderator of the assumed relationship between trait mindfulness and pain adaptation. We had the following hypotheses:1) There is a positive correlation between trait mindfulness and the degree of pain adaptation, a negative correlation between extraversion and the degree of pain adaptation, and a negative correlation between trait mindfulness and extraversion. 2) Higher levels of trait mindfulness predict greater degrees of pain adaptation (larger difference between the first and last trial of pain intensity ratings) and higher levels of extraversion predict lower degrees of pain adaptation. 3) The relationship between trait mindfulness and pain adaptation is moderated by individual levels of extraversion, due to significant interactions between the two traits.

**2. Materials and Method**

The study complies with the latest revision of the Declaration of Helsinki (DRKS-ID: DRKS00032271). Experimental procedures were approved by the local ethics committee of the Medical Faculty at Kiel University (AZD482/22). All participants were informed about the study and provided written consent.

***2.1. Participants***

Fifty-two healthy volunteers (mean age = 23.29 ± 2.052 years; 19 males; 33 females; sample characterization, see Table 1) participated in the study. They were recruited via flyers and online advertisements.

Inclusion criteria were: German native speakers, right-handed, age range 20 to 30 years (to control for a potential confounding effect of age).

Exclusion criteria were: any prior mindfulness-based training (to control for a potential confounding effect due to experiences with mindfulness traditions and trainings), any psychiatric, neurological, or internal diseases, as well as chronic or subacute pain, current or former drug addiction, intake of regular medication (except for hormone contraception) especially analgesic drugs, or central nervous system-effective medications, pregnancy, IQ below 80 or former meditation experience (excluding yoga) within the past 8 weeks. For these, participants underwent a pregnancy test and the basic intelligence was tested by the revised German Culture Fair Intelligence Test 2 (CFT-20-R) (Weiß, Albinus, & Arzt, 2006) and the dominant hand by the Edinburgh Inventory (Oldfield, 1971). Other screenings were conducted primarily through structured interviews and participants’ self-reports.

***2.2. Psychometric Assessments and Experimental Condition***

Questionnaires of trait mindfulness and extraversion were completed online, using the SoSci tool (Leiner, 2021), before joining the experimental pain adaptation task in the laboratory.

2.2.1 Trait Mindfulness

We assessed trait mindfulness using the mean of the total scores of the Mindful Attention and Awareness Scale, German Version (MAAS) (Michalak, 2008), with higher scores indicating a higher level of trait mindfulness. The MAAS comprises 15 self-referential statements, e.g., ‘‘I find it difficult to stay focused on what is happening in the present moment ‘‘, measured on a 6-point scale (1 = Almost always, 6 = Almost never).

2.2.2 Extraversion

We assessed extraversion using the mean of the respective subscale of the NEO Five-Factor Inventory, German Version (NEO-FFI) (Costa & McCrae, 2008), with higher scores indicating a higher level of extraversion. The NEO-FFI includes 12 items for extraversion, e.g., ‘‘I like having lots of people around me ‘‘, measured on a 5-point scale (0 =Strong rejection, 4 = Strong agreement).

2.2.3 Experimental procedure

Pain adaptation was assessed using self-rate information from an experimental pain stimulation session ranging on a visual analog scale (VAS) from zero to 100.

2.2.3.1 Pain Stimulation Session

A standardized protocol was conducted to identify the temperature used as the pain stimulus in the subsequent pain rating tasks for each participant (Rolke et al., 2006). To this end, thermal stimuli were applied by using a TSA-2001 thermal stimulator (Medoc Advanced Medical Systems, Haifa, Israel) with a 30 mm × 30 mm flat thermode to the ventral side of the left forearm. The temperature of thermode was increased from baseline (32°C) to maximum (49°C) with a rate of 1.5°C/s while the subject had the task of resetting the temperature by button-press when reaching certain subjective pain sensations. Participants were instructed to stop the increasing thermal stimulus as soon as the pain reached a subjective pain intensity of 80 out of 100 on a verbally communicated 100-point scale with evenly distributed integers from 0 (no pain) to 100 (unbearable pain). This procedure was repeated 10 times. Subsequently, the pain stimulus for the pain rating task was defined as the average temperatures of the last 5 trials. In case the maximum temperature (49°C) did not reach the participants’ 80-level pain intensity, 49°C was set as the pain stimulus temperature for the pain adaptation task.

The pain adaptation task consisted of 20 consecutive trials. In each trial the temperature of the thermode was increased from the baseline (32°C) to the participants' target temperature in two seconds, remaining at that level for 10 seconds before decreasing to the baseline. Subsequently, the participants were instructed to rate the perceived pain intensity using a VAS from 0 to 100, which was presented for 10 s, followed by a 10 s relaxation period.

Pain adaptation was measured by calculating the difference in pain intensity ratings between the first and the last trial, with a larger difference indicating a higher degree of pain adaptation. Due to technical reasons, data only for 15 trials were available. However, as summarized in a recent review, the number of trials in previous studies investigating pain adaptation has ranged from 3 to 28 (van der Miesen et al., 2024), and the main pain adaptation process (habituation or sensitization) has been found specifically within 10 trials (Edwards & Fillingim, 2001; Edwards, Smith, Stonerock, & Haythornthwaite, 2006; Koenen et al., 2017). Therefore, our data still capture a valid window, and since we also wanted to include any potential re-adaptation processes within our analyses, we analyzed the 15 trials from our procedure. As a supplement, we also conducted the same data analyses under 10-trial conditions (see Supplementary Material).

***2.4. Data Analyses***

To examine the relationship between trait mindfulness and extraversion on pain adaptation as well as between both traits, we performed correlation analyses with all variables and then the partial correlation analyses controlling for sex (considering the imbalance in the number of male and female participants in our sample). Moreover, we performed a hierarchical regression analysis, with pain adaptation as the outcome and the trait variables (mindfulness and extraversion) as the predictors. Those were entered in the first step, and then in the second step, sex was entered into the model as a covariate to control for potential effects of sex on the prediction of pain adaptation by trait mindfulness and extraversion. To examine potential trait-related moderation effects, the product of trait mindfulness and extraversion was entered into the model as an interaction term in the last step and the simple slope analysis was applied to illustrate the relationship between trait mindfulness and pain adaptation at higher and lower levels of extraversion (i.e., one standard deviation above and below the mean of the moderator). In addition, the Johnson-Neyman technique was employed to identify specific ranges of extraversion where the effect of trait mindfulness on pain adaptation was significant. The assumptions of the regression model (normal distribution, variance homogeneity, autocorrelation, multicollinearity, and casewise diagnostics for influential outliers) were tested, with the results generally supporting the use of regression analysis. Detailed results of these tests are presented in the supplementary material.

All of the above analyses were performed in SPSS 21.0 (IBM Corp., Armonk, NY, U.S.A), including Hayes PROCESS 4.2 macro (Hayes, 2017). The Johnson-Neyman plot was created using the ggplot2 package in R (Wickham & Wickham, 2016). We applied a p-value < 0.05 (all tests were two-sided).

**3. RESULTS**

***3.1*** ***Correlations between trait Mindfulness, Extraversion, and Pain Adaptation.***

Table 1 shows the results of the correlation analysis. Neither the correlation between trait mindfulness and the degree of pain adaptation (r = -0.009, p = 0.949) nor the correlation between extraversion and the degree of pain adaptation (r = 0.039, p = 0.784) reached a statistically significant level. However, trait mindfulness was positively correlated with extraversion (r = 0.347, p = 0.012). Additionally, the correlation between sex and trait mindfulness (r = 0.356, p = 0.010), as well as the correlation between sex and pain stimulus temperature (r = 0.277, p = 0.047) are both significant.

When controlling for the sex, the correlations between trait mindfulness, extraversion, and pain adaptation remained the same, meaning that trait mindfulness and adaptation (r = -0.036, p = 0.803) and extraversion and pain adaptation (r = 0.031, p = 0.826) were still not significantly correlated, but trait mindfulness and extraversion showed a significant correlation (r = 0.330, p = 0.018) (see also Table 2).

-Insert Table 1 here-

-Insert Table 2 here-

***3.2*** ***The Predictive Value of Trait Mindfulness and Extraversion for Pain Adaptation.***

Table 3 shows the results of the hierarchical regression analysis. As shown in Model 1, neither the level of trait mindfulness (β = -0.026, p = 0.866) nor the level of extraversion (β = 0.048, p = 0.754) significantly predicted the degree of pain adaptation. As shown in Model 2, when controlling for sex, neither the level of trait mindfulness (β = -0.055, p = 0.735) nor the level of extraversion (β = 0.049, p = 0.752) significantly predicts the degree of pain adaptation.

***3.3*** ***The Moderation of the Relationship between Trait Mindfulness and Pain Adaptation by Extraversion.***

As shown in Model 3 (see Table 3), there was a significant interaction between trait mindfulness and extraversion (β = -0.528, p < 0.001).

Applying the simple slope analysis (see Figure 1), we see that at a lower level of extraversion, a higher level of trait mindfulness predicted a larger degree of pain adaptation (b = 9.307, p = 0.017). At a higher level of extraversion, a higher level of trait mindfulness predicted a lesser degree of pain adaptation (b = -10.449, p = 0.005), while at a medium level of extraversion, a higher level of trait mindfulness did not significantly predict a lesser degree of pain adaptation (b = -0.571, p = 0.841).

As shown in Figure 2, the effect of trait mindfulness was significant at values of extraversion either below -0.333, or above 0.251. Outside of these ranges, the effect was not significant.

-Insert Table 3 here-

-Insert Figure 1 here-

-Insert Figure 2 here-

**4. DISCUSSION**

In the present study, we aimed to investigate the potential impact of trait mindfulness and extraversion as well as their interaction on an experimental pain adaptation process. We found that trait mindfulness and extraversion were positively correlated, while neither trait mindfulness nor extraversion alone showed a significant association with pain adaptation. These effects were independent of sex, which still significantly correlated with trait mindfulness and pain stimulus temperature. However, we observed a significant interaction between trait mindfulness and extraversion in relation to pain adaptation, showing that the effect of mindfulness on pain adaptation is moderated by the level of extraversion. Specifically, higher trait mindfulness was associated with greater pain adaptation when extraversion levels were low (below -0.333, the value was centered by the mean), but with lesser pain adaptation when extraversion levels were high (above 0.251, the value was centered by the mean).

Contrary to our hypothesis, trait mindfulness was neither significantly correlated with nor a significant predictor of pain adaptation. This is inconsistent with previous studies that suggested a link between trait mindfulness and either facilitation (Harrison et al., 2023) or impairment of the pain adaptation process (Ginzburg et al., 2015). However, while neither trait mindfulness nor extraversion significantly predicted pain adaptation on their own, their interaction was significant. The results of simple slope analysis and the Johnson-Neyman technique showed that trait mindfulness significantly predicted pain adaptation at both lower and higher levels of extraversion but in opposite directions. This suggests that the influence of trait mindfulness on pain adaptation is dependent on levels of extraversion, which might explain the mixed results in earlier research. Similarly, the level of extraversion was neither significantly correlated with nor a predictor of pain adaptation, but this could be explained by the significant interaction between extraversion and mindfulness as well. Our study found that extraversion was correlated with trait mindfulness and moderated the relationship between trait mindfulness and pain adaptation, which is consistent with our hypothesis. However, contrary to our expectations, the moderating effect of extraversion was not based on a negative correlation with trait mindfulness; rather, a positive correlation was observed, suggesting that individuals with higher extraversion are also more likely to have higher trait mindfulness, which is also partly in line with previous studies (Banfi & Randall, 2022; Giluk, 2009; Hanley & Garland, 2017). This might be attributable to both traits being associated with some positive factors, e.g., subjective well-being and positive emotions (Brown et al., 2007). Nevertheless, this combination might be detrimental in the context of pain adaptation, leading to lesser pain adaptation.

While our study was performed in a non-clinical sample, in patients with chronic pain, studies have shown that individuals with higher trait mindfulness experience less distress and better well-being (Miniati et al., 2023), and mindfulness training is often seen as promising for pain management (Braden et al., 2016; Day et al., 2021; Mioduszewski et al., 2020; Parisi, Hanley, & Garland, 2022; Smith et al., 2021). However, our findings suggest that mindfulness training may be more beneficial for individuals with lower extraversion in managing pain, while this may not hold for individuals with higher extraversion. Future clinical studies are needed to explore this further.

Interestingly, the moderating effect of extraversion changes the direction of how trait mindfulness influences pain adaption. A possible explanation is that even though both mindfulness (Li, Yang, Zhang, Xu, & Cai, 2021; Petranker & Eastwood, 2021) and extraversion (Lou et al., 2016; Yu et al., 2016) are attention-related traits, the strategies for pain adaptation driven by higher trait mindfulness and higher extraversion might contradict each other. Specifically, for pain relief or adaptation, higher trait mindfulness may achieve by lessening cognitive interference when remaining attention on pain (Gard et al., 2012; Lu, Moliadze, & Nees, 2023; Wang, 2015; Zeidan et al., 2018; Zeidan & Vago, 2016), while higher extraversion might drive a shift in attention away from pain (Yu et al., 2016). Therefore, for pain management, mindfulness-based training may be particularly beneficial for individuals with lower levels of extraversion. In contrast, individuals with higher levels of extraversion might benefit more from training that focuses on improving their ability to effectively shift attention away from the painful area, rather than enhancing the capacity to maintain attention on it with acceptance and a non-reactive attitude. However, these are only potential clinical applications of current findings, which remain to be further examined in future clinical studies with mindfulness-based interventions and other distraction-based interventions.

There are some limitations of the current study. First, while 20 trials were planned for the experimental condition; however, technical issues reduced the number of usable trials to 15. While this reduction might introduce bias, it should be noted that previous studies have also used 15 trials. To still evaluate the impact of this limitation, we conducted additional analyses with an even smaller number of trials (see Supplementary material). The results were generally consistent, although the prediction of trait mindfulness on pain adaptation did not reach significance in the low extraversion condition. Therefore, future studies should focus on different time windows and also integrate information on additional traits. Second, the use of thermal stimuli at a single intensity level (80 out of 100) and the inclusion (aged from 20 to 30) and exclusion criteria (healthy individuals without mindfulness-based training experience) implemented to control for confounding factors warrant caution when generalizing our findings to other stimulus conditions or populations. In addition, the assessment of trait mindfulness is based on the MAAS, which is unidimensional, i.e., focuses on the attention aspect of mindfulness (Bergomi, Tschacher, & Kupper, 2013), based on which there may be inconsistencies between our findings and those from other studies. Therefore, future studies should consider validating these results with other questionnaires measuring multiple dimensions of mindfulness (e.g., the Five Facet Mindfulness Questionnaire, FFMQ) (Carpenter et al., 2019) or additional measures such as neurophysiological indicators, which would also provide the possibility to explore whether specific facets of trait mindfulness and extraversion are differentially associated with pain adaptation. Furthermore, based on our literature combing and research questions, the significant interaction between trait mindfulness and extraversion was elaborated as a moderating effect of extraversion on the relationship between trait mindfulness and pain adaptation. However, trait mindfulness as the moderator in the relationship between extraversion and pain adaptation may also be inferred from the same results. Nevertheless, this would not fundamentally change our conclusions about the significant interaction of the two traits in influencing pain adaptation. Finally, the main focus and interest of this study is the relationship between trait mindfulness and extraversion, as well as their influence on pain adaptation. However, it is important to acknowledge that the pain adaptation process may be affected not only by these two traits but also by other traits or psychological factors. Future research integrating a broader range of potential determinants would be valuable in developing a more comprehensive predictive model of the pain adaptation process where huge individual differences could exist (see Figure 3).

-Insert Figure 3 here-

**5. Conclusion**

Our study highlights the correlations between traits and their interactions in influencing pain habituation, indicating that the degree to which individuals with higher levels of trait mindfulness experience greater pain adaptation depends on their levels of extraversion. Given that altered pain adaptation has been shown and discussed as a potential risk factor for pain chronicity, our findings could suggest that these traits could serve as protective factors in the development of chronic pain and imply that mindfulness-based interventions might be especially effective for individuals with certain traits. Future research should further investigate these implications to improve pain management strategies.

**Acknowledgments**

**Funding:** We thank the China Scholarship Council (CSC) for supporting this study, which is a scholarship for Chen Lu, the first author of this paper.

**Author Contributions:** All authors contributed extensively to the work presented in this paper.

Study conception and design: Chen Lu, Frauke Nees, Vera Moliadze;

Material preparation, data collection: Chen Lu, Vera Moliadze, Nele Berner, Lena Hagel, Nils Jannik Heukamp;

Data analysis: Chen Lu;

Writing - original draft preparation: Chen Lu, Frauke Nees;

Writing - review and editing: Chen Lu, Frauke Nees, Vera Moliadze, Nils Jannik Heukamp;

Funding acquisition: Frauke Nees, Chen Lu (scholarship from CSC);

Supervision: Frauke Nees.

All authors discussed the results and implications and commented on the manuscript at all stages. All authors reviewed the manuscript. All authors have approved the submitted version.

**Conflict of Interest:** All authors declare that they have no competing interests.

**Ethics/Ethical Approval:** The study complies with the latest revision of the Declaration of Helsinki (DRKS-ID: DRKS00032271). Experimental procedures were approved by the local ethics committee of the Medical Faculty at Kiel University (AZD482/22). All participants were informed about the study and provided written consent.

**Data Availability:** Due to ethical restrictions, data are available from the corresponding author upon reasonable request.

References

Banfi, J. T., & Randall, J. G. (2022). A meta-analysis of trait mindfulness: Relationships with the big five personality traits, intelligence, and anxiety. *Journal of Research in Personality, 101*, 104307.

Bergomi, C., Tschacher, W., & Kupper, Z. (2013). The assessment of mindfulness with self-report measures: Existing scales and open issues. *Mindfulness, 4*, 191-202.

Bingel, U., Schoell, E., Herken, W., Büchel, C., & May, A. (2007). Habituation to painful stimulation involves the antinociceptive system. *Pain, 131*(1-2), 21-30.

Braden, B. B., Pipe, T. B., Smith, R., Glaspy, T. K., Deatherage, B. R., & Baxter, L. C. (2016). Brain and behavior changes associated with an abbreviated 4‐week mindfulness‐based stress reduction course in back pain patients. *Brain and behavior, 6*(3), e00443.

Brown, K. W., Ryan, R. M., & Creswell, J. D. (2007). Mindfulness: Theoretical foundations and evidence for its salutary effects. *Psychological inquiry, 18*(4), 211-237.

Carpenter, J. K., Conroy, K., Gomez, A. F., Curren, L. C., & Hofmann, S. G. (2019). The relationship between trait mindfulness and affective symptoms: A meta-analysis of the Five Facet Mindfulness Questionnaire (FFMQ). *Clinical psychology review, 74*, 101785.

Ciere, Y., Snippe, E., Padberg, M., Jacobs, B., Visser, A., Sanderman, R., & Fleer, J. (2019). The role of state and trait positive affect and mindfulness in affective reactivity to pain in chronic migraine. *Health Psychology, 38*(1), 94.

Costa, P. T., & McCrae, R. R. (2008). The revised neo personality inventory (neo-pi-r). *The SAGE handbook of personality theory and assessment, 2*(2), 179-198.

Day, M. A., Jensen, M. P., Ehde, D. M., & Thorn, B. E. (2014). Toward a theoretical model for mindfulness-based pain management. *The journal of pain, 15*(7), 691-703.

Day, M. A., Matthews, N., Mattingley, J. B., Ehde, D. M., Turner, A. P., Williams, R. M., & Jensen, M. P. (2021). Change in brain oscillations as a mechanism of mindfulness-meditation, cognitive therapy, and mindfulness-based cognitive therapy for chronic low back pain. *Pain Medicine, 22*(8), 1804-1813.

Day, M. A., & Thorn, B. E. (2016). The mediating role of pain acceptance during mindfulness-based cognitive therapy for headache. *Complementary Therapies in Medicine, 25*, 51-54.

Defrin, R., Riabinin, M., Feingold, Y., Schreiber, S., & Pick, C. G. (2015). Deficient pain modulatory systems in patients with mild traumatic brain and chronic post-traumatic headache: implications for its mechanism. *Journal of neurotrauma, 32*(1), 28-37.

Diener, E., Suh, E. M., Lucas, R. E., & Smith, H. L. (1999). Subjective well-being: Three decades of progress. *Psychological bulletin, 125*(2), 276.

Edwards, R. R., & Fillingim, R. B. (2001). Effects of age on temporal summation and habituation of thermal pain: clinical relevance in healthy older and younger adults. *The journal of pain, 2*(6), 307-317.

Edwards, R. R., Smith, M. T., Stonerock, G., & Haythornthwaite, J. A. (2006). Pain-related catastrophizing in healthy women is associated with greater temporal summation of and reduced habituation to thermal pain. *The Clinical journal of pain, 22*(8), 730-737.

Ferracuti, S., & De Carolis, A. (2005). Relationships among Eysenck's extraversion, Rorschach's Erlebnistypus, and tolerance of experimental tonic pain (Cold Water Pressor Test). *Perceptual and motor skills, 100*(1), 237-248.

Gard, T., Hölzel, B. K., Sack, A. T., Hempel, H., Lazar, S. W., Vaitl, D., & Ott, U. (2012). Pain attenuation through mindfulness is associated with decreased cognitive control and increased sensory processing in the brain. *Cereb Cortex, 22*(11), 2692-2702. doi:10.1093/cercor/bhr352

Giluk, T. L. (2009). Mindfulness, Big Five personality, and affect: A meta-analysis. *Personality and Individual Differences, 47*(8), 805-811.

Ginzburg, K., Tsur, N., Karmin, C., Speizman, T., Tourgeman, R., & Defrin, R. (2015). Body awareness and pain habituation: the role of orientation towards somatic signals. *Journal of Behavioral Medicine, 38*, 876-885.

Grouper, H., Eisenberg, E., & Pud, D. (2021). More insight on the role of personality traits and sensitivity to experimental pain. *Journal of pain research*, 1837-1844.

Hanley, A. W., & Garland, E. L. (2017). The mindful personality: A meta-analysis from a cybernetic perspective. *Mindfulness, 8*, 1456-1470.

Harrison, R., Salomons, T. V., van Reekum, C. M., Adams, G., & Gandhi, W. (2023). Temporal dynamics of hippocampal activity predict stable patterns of sensitization or habituation to noxious stimulation across sessions. *bioRxiv*, 2023.2009. 2006.555302.

Harrison, R., Zeidan, F., Kitsaras, G., Ozcelik, D., & Salomons, T. V. (2019). Trait mindfulness is associated with lower pain reactivity and connectivity of the default mode network. *The Journal of Pain, 20*(6), 645-654.

Hayes, A. F. (2017). *Introduction to mediation, moderation, and conditional process analysis: A regression-based approach*: Guilford publications.

HE, Y.-Y., YUAN, J.-J., WU, Z.-L., & LI, H. (2008). The valence strength of positive stimuli modulates the attention in extraverts study. *Acta Psychologica Sinica, 40*(11), 1158.

Kiken, L. G., Garland, E. L., Bluth, K., Palsson, O. S., & Gaylord, S. A. (2015). From a state to a trait: Trajectories of state mindfulness in meditation during intervention predict changes in trait mindfulness. *Personality and Individual differences, 81*, 41-46.

Koenen, L. R., Icenhour, A., Forkmann, K., Pasler, A., Theysohn, N., Forsting, M., . . . Elsenbruch, S. (2017). Greater fear of visceral pain contributes to differences between visceral and somatic pain in healthy women. *Pain, 158*(8), 1599-1608.

Kusko, D. (2023). Higher Trait Mindfulness Is Associated With Less Experimental Pain In Individuals With Knee Osteoarthritis. *The Journal of Pain, 24*(4), 57-58.

Leiner, D. (2021). SoSci Survey (Version 3.1. 06). 2019. *URL: <https://www>. soscisurvey. de [accessed 2021-05-01]*.

Li, Y., Yang, N., Zhang, Y., Xu, W., & Cai, L. (2021). The relationship among trait mindfulness, attention, and working memory in junior school students under different stressful situations. *Frontiers in Psychology, 12*, 558690.

Lou, Y., Meng, X., Yang, J., Zhang, S., Long, Q., & Yuan, J. (2016). The impact of extraversion on attentional bias to pleasant stimuli: neuroticism matters. *Experimental brain research, 234*, 721-731.

Lu, C., Moliadze, V., & Nees, F. (2023). Dynamic processes of mindfulness-based alterations in pain perception. *Frontiers in Neuroscience, 17*.

Lukaszewski, A. W. (2020). Extraversion. *Encyclopedia of personality and individual differences*, 1514-1519.

Michalak, J. (2008). Mindful Attention and Awareness Scale, deutsche Version (MAAS). *Zeitschrift für Klinische Psychologie und Psychotherapie, 37*(3), 200-208.

Miniati, M., Orrù, G., Paroli, M., Cinque, M., Paolicchi, A., Gemignani, A., . . . Palagini, L. (2023). Patients with Chronic Pain: Are Mindfulness Traits Protective Against Distress, Anxiety and Depression? *Clinical Neuropsychiatry, 20*(5), 429.

Mioduszewski, O., Hatchard, T., Fang, Z., Poulin, P., Khoo, E.-L., Romanow, H., . . . Browne, N. (2020). Breast cancer survivors living with chronic neuropathic pain show improved brain health following mindfulness-based stress reduction: a preliminary diffusion tensor imaging study. *Journal of Cancer Survivorship, 14*, 915-922.

Oldfield, R. C. (1971). The assessment and analysis of handedness: the Edinburgh inventory. *Neuropsychologia, 9*(1), 97-113.

Parisi, A., Hanley, A. W., & Garland, E. L. (2022). Mindfulness-Oriented Recovery Enhancement reduces opioid craving, pain, and negative affect among chronic pain patients on long-term opioid therapy: An analysis of within-and between-person state effects. *Behaviour Research and Therapy, 152*, 104066.

Petranker, R., & Eastwood, J. D. (2021). Sitting with it: An investigation of the relationship between trait mindfulness and sustained attention. *Consciousness and cognition, 90*, 103101.

Phillips, J. M., & Gatchel, R. J. (2000). Extraversion–introversion and chronic pain.

Rolke, R., Baron, R., Maier, C. a., Tölle, T., Treede, R.-D., Beyer, A., . . . Bötefür, I. (2006). Quantitative sensory testing in the German Research Network on Neuropathic Pain (DFNS): standardized protocol and reference values. *PAIN®, 123*(3), 231-243.

Shapiro, S. L., Brown, K. W., Thoresen, C., & Plante, T. G. (2011). The moderation of mindfulness‐based stress reduction effects by trait mindfulness: results from a randomized controlled trial. *Journal of clinical psychology, 67*(3), 267-277.

Smith, A., Leeming, A., Fang, Z., Hatchard, T., Mioduszewski, O., Schneider, M., . . . Poulin, P. (2021). Mindfulness-based stress reduction alters brain activity for breast cancer survivors with chronic neuropathic pain: preliminary evidence from resting-state fMRI. *Journal of Cancer Survivorship, 15*, 518-525.

van der Miesen, M. M., Joosten, E. A., Kaas, A. L., Linden, D. E., Peters, J. C., & Vossen, C. J. (2024). Habituation to pain: self-report, electroencephalography, and functional magnetic resonance imaging in healthy individuals. A scoping review and future recommendations. *Pain, 165*(3), 500-522.

van der Miesen, M. M., Vossen, C. J., & Joosten, E. A. (2023). Habituation to Pain in Patients with Chronic Pain: Clinical Implications and Future Directions. *Journal of Clinical Medicine, 12*(13), 4305.

Vassend, O. B., Røysamb, E., & Nielsen, C. S. (2013). Five-factor personality traits and pain sensitivity: A twin study. *PAIN®, 154*, 722-728.

Wade, J. B., & Price, D. D. (2000). Nonpathological factors in chronic pain: Implications for assessment and treatment.

Wang, Y. (2015). Mindfulness training improves acceptance of pain. *Chinese Journal of Clinical Psychology, 23*(3), 567-570.

Weiß, R., Albinus, B., & Arzt, D. (2006). *Grundintelligenztest Skala 2-Revision (CFT 20-R)*: Hogrefe.

Wickham, H., & Wickham, H. (2016). *Data analysis*: Springer.

Yu, Y., Yang, Q., Li, J., Xu, Y., Liu, B., Peng, L., . . . Li, M. (2016). Mediating and moderating effects of attentional control on the relationship between personality traits and attentional bias among Chinese medical students. *Psychiatry Research, 246*, 113-118.

Zeidan, F., Salomons, T., Farris, S. R., Emerson, N. M., Adler-Neal, A., Jung, Y., & Coghill, R. C. (2018). Neural mechanisms supporting the relationship between dispositional mindfulness and pain. *Pain, 159*(12), 2477-2485.

Zeidan, F., & Vago, D. R. (2016). Mindfulness meditation–based pain relief: a mechanistic account. *Annals of the New York Academy of Sciences, 1373*(1), 114-127.

**Table Legends**

**Table 1. Correlations of Variables.**

***** p < 0.05, ****** p < 0.01

Abbreviations: M, Mean; MAAS, Mindful Attention and Awareness Scale; NEO-FFI, NEO Five Factors Inventory; SD, Standard Deviation.

**Table 2. Correlations of Variables When Controlling for Sex.**

***** p < 0.05

Abbreviations: M, Mean; MAAS, Mindful Attention and Awareness Scale; NEO-FFI, NEO Five Factors Inventory; SD, Standard Deviation.

**Table 3. Hierarchical Regression Analysis Predicting Pain Adaptation from Trait Mindfulness, Extraversion, and the Mindfulness × Extraversion Interaction.**

****** p **< 0.01, ***** p **< 0.001**

All continuous independent variables were centered (original values minus mean) before being entered into the models. Outside the brackets are the B values (unstandardized coefficient) and inside the brackets are the β values (standardized coefficient).

**Figure Legends**

**Figure 1.**

**The Relationship between Trait Mindfulness and Pain Adaptation at Three Levels of Extraversion.**

All continuous independent variables were centered (original values minus mean) before being entered into the models and the units for each variable were one point. The pain adaptation was assessed by the difference between the first trials and the fifteenth trials of ratings. The relationship between trait mindfulness and pain adaptation was examined on three levels of extraversion and levels of extraversion were defined by one standard deviation below the mean, mean, and one standard deviation above the mean.

**Figure 2.**

**Johnson-Neyman plot of the conditional effect of Trait mindfulness on Pain Adaptation across Extraversion.**

All continuous independent variables were centered (original values minus mean) before being entered into the models and the units for each variable were one point. The pain adaptation was assessed by the difference between the first trials and the fifteenth trials of ratings. The grey shaded area indicates the confidence interval (CI), and the light blue shaded area indicates the significance area.

**Figure 3.**

**The visualization of the pain adaptation results according to the characteristic information.**

The pain adaptation was assessed by the difference between the first trials and the fifteenth trials of ratings. A. The degree of pain adaptation for each participant. B. The degree of pain adaptation for male and female participants. C. The degree of pain adaptation for participants with low, moderate, and high levels of trait mindfulness. The value of trait mindfulness was centered (original values minus mean) and then the levels of trait mindfulness were defined by lower than one standard deviation below the mean (low), from one standard deviation below the mean to one standard deviation above the mean (moderate), and higher than one standard deviation above the mean (high). D. The degree of pain adaptation for participants with low, moderate, and high levels of extraversion (high). The value of extraversion was centered (original values minus mean) and then the levels of extraversion were defined by lower than one standard deviation below the mean (low), from one standard deviation below the mean to one standard deviation above the mean (moderate), and higher than one standard deviation above the mean (high). In these boxplots, the box represents the interquartile range (IQR), spanning from the first quartile (Q1) to the third quartile (Q3). A horizontal line within the box marks the median, while the whiskers extend from the box to capture the spread of the data, up to 1.5 times the IQR from Q1 and Q3.

**Tables**

| **Table 1. Correlations of Variables.** | | | | | | | |
| --- | --- | --- | --- | --- | --- | --- | --- |
| **Variables** | **M ± SD** | **Observed Range** | **Trait Mindfulness (MAAS)** | **Extraversion (NEO-FFI)** | **Pain Adaptation** | **Pain Stimulus Temperature** | **Sex** |
| **Trait Mindfulness (MAAS)** | **3.76 ± 0.739** | **2.20 ~ 4.87** | **1** |  |  |  |  |
| **Extraversion (NEO-FFI)** | **2.43 ± 0.443** | **1.50 ~ 3.33** | **0.347^*^** | **1** |  |  |  |
| **Pain Adaptation** | **3.67 ± 14.961** | **-32.41 ~ 49.07** | **-0.009** | **0.039** | **1** |  |  |
| **Pain Stimulus Temperature** | **46.64 ± 1.921** | **38.80 ~ 49.00** | **0.103** | **0.072** | **-0.063** | **1** |  |
| **Sex** | **female** = **0**  **male** = **1** |  | **0.356^**^** | **0.114** | **0.068** | **0.277^*^** | **1** |
| **Age** | **23.29 ± 2.052** | **20 ~ 30** | **-0.163** | **-0.002** | **-0.055** | **-0.164** | **0.148** |

***** p < 0.05, ****** p < 0.01

Abbreviations: M, Mean; MAAS, Mindful Attention and Awareness Scale; NEO-FFI, NEO Five Factors Inventory; SD, Standard Deviation.

| **Table 2. Correlations of Variables When Controlling for Sex.** | | | | | |
| --- | --- | --- | --- | --- | --- |
| **Variables** | | **Trait Mindfulness (MAAS)** | **Extraversion (NEO-FFI)** | **Pain Adaptation** | **Pain Stimulus Temperature** |
| **Sex (Control Variable)** | **Trait Mindfulness (MAAS)** | **1** |  |  |  |
|  | **Extraversion (NEO-FFI)** | **0.330*** | **1** |  |  |
|  | **Pain Adaptation** | **-0.036** | **0.031** | **1** |  |
|  | **Pain Stimulus Temperature** | **0.005** | **0.042** | **-0.085** | **1** |
|  | **Age** | **-0.233** | **-0.020** | **-0.066** | **-0.216** |

***** p < 0.05

Abbreviations: M, Mean; MAAS, Mindful Attention and Awareness Scale; NEO-FFI, NEO Five Factors Inventory; SD, Standard Deviation.

**Table 3. Hierarchical Regression Analysis Predicting Pain Adaptation from Trait Mindfulness, Extraversion, and the Mindfulness × Extraversion Interaction.**

| **Variable** | **Model 1 (Predictors)** | **Model 2 (Predictors and Control Variable)** | **Model 3 (Moderation Model)** |
| --- | --- | --- | --- |
| **Sex (female = 0, male = 1)** |  | **2.536 (0.082)** | **4.158 (0.135)** |
| **Trait Mindfulness** | **-0.522 (-0.026)** | **-1.122 (-0.055)** | **-0.583 (-0.029)** |
| **Extraversion** | **1.620 (0.048)** | **1.648 (0.049)** | **1.218 (0.036)** |
| **Trait Mindfulness × Extraversion** |  |  | **-22.301 (-0.528) ***** |
| **R²** | **0.002** | **0.008** | **0.283** |
| **ΔR²** | **0.002** | **0.006** | **0.275** |
| **F** | **0.052** | **0.130** | **4. 630**** |
| **df** | **2, 49** | **3, 48** | **4, 47** |

****** p **< 0.01, ***** p **< 0.001**

All continuous independent variables were centered (original values minus mean) before being entered into the models. Outside the brackets are the B values (unstandardized coefficient) and inside the brackets are the β values (standardized coefficient).
